# 

**Published:** 1875-11

**Authors:** 


### ANNOUNCEMENTS FOR THE MONTH.

### MONDAYS.

SOCIETIES.
*Mondays, Nov.* 1 and 15--Chicago Med. Society, regular meetings at Gault House, 8 P.M.
*Mondays, Nov.* 8 and 22 — Chicago Society of Physicians and Surgeons, regular meetings at Grand Pacific, 8 P. M.

CLINICS. *Every Monday.*
At Eye and Ear Infirmary, (Peoria and Adams Sts.) 2 P. M.—Prof. Holmes.
At Chicago College, 2 P. M. *Gynecological*—Prof. Merriman.
At Central Dispensary (239 W. Van Buren St.), 2 P.M., *Gynecological*—Dr. Adolphus.
At Mercy Hospital, 2 P. M. *Medical*—Prof. Johnson

LECTURES *Every Monday.* At Rush College (18th and Arnold Sts.), 8½ to 12½ o'clock—Profs. Gunn, Miller, Freer and Powell; 4 to 6—Profs. Lyman and Etheridge. At Chicago College, 8½ to 12½—Profs. Jewell, Isham, Hyde and Bond; 3 to 6—Profs. Davis and Nelson, Andrews and Quine, and Roler. At Womans' Hospital College, 9 to 12—Profs. Thompson, Bogue and Blake; 3 to 6—Profs. Paoli, Stevenson and MacDonald.

### TUESDAYS.

SOCIETIES.
*Tuesday, Nov.* 9th — Academy of Sciences, regular meeting, 8 P. M. (263 Wabash Av).

CLINICS. *Every Tuesday.*
At County Hospital, 2 P. M., *Medical*—Prof. Ross; 3 P. M., *Surgical*—Prof. Powell.
At Chicago College, 2 P. M., *Gynecological*—Prof. Roler.
At Mercy Hospital, 2 P. M., *Medical*—Prof. Hollister.

LECTURES. *Every Tuesday.*
At Rush College, 8½ to 12½—Profs. Gunn, Miller, Allen and Parkes; 4—Prof. Lyman. At Chicago College, 8½ to 12½—Profs. Jewell, Isham, Merriman and Bond; 3 to 6—Profs. Davis and Nelson, Andrews and Hatfield, and Byford. At Woman's Hosp. Coll., 9 to 12—Profs. Marguerat, Bartlett and Fitch;—3 to 6, Profs. Delafontaine, Curtis and MacDonald.

### WEDNESDAYS.

CLINICS. *Every Wednesday.*
At County Hospital, 2 P. M., *Ophthalmological*—Dr. Montgomery; 3 P. M., *Gynecological*—Prof. Fitch.
At Chicago College, 2 P. M., *Gynecological*—Prof. Nelson.
At Woman's Dispensary (229, 30th St.), 11 A. M., *Gynecological*—Drs. Hurlbut and Jackson; 1 P. M., *Electrical*—Dr. P. S. Hayes.
At Mercy Hospital, 2 P. M., *Surgical*—Prof. Andrews.

LECTURES. *Every Wednesday.*
At Rush College, 8½ to 12½—Profs. Hay, Freer, Allen and Parkes: 4 to 6—Profs. Lyman and Etheridge. At Chicago College, 8½ to 12½—Profs. Hollister, Isham, Hyde and Bond; 3 to 6—Profs. Davis and Curtis, Jones and Quine, and Roler. At Woman's Hosp. Coll., 9 to 12—Profs. Dyas, Earle and Thompson; 3 to 5—Profs. Paoli and Stevenson.

### THURSDAYS.

CLINICS. *Every Thursday.*
At Eye and Ear Infirmary, 2 P. M.—Prof. Holmes.
At Chicago College, 2 P. M., *Gynecological*—Prof. Merriman.
At Central Dispensary, 2 P. M., *Gynecological*—Dr. Adolphus.
At Mercy Hospital, 2 P. M., *Medical*—Prof. Johnson.

LECTURES. *Every Thursday.*
At Rush College, 8½ to 12½—Profs. Gunn, Miller, Allen and Parkes; 4 to 6—Profs. Ross and Lyman. At Chicago College, 8½ to 12½—Profs. Hollister, Isham, Merriman and Bond; 3 to 6—Profs. Davis and Nelson, Andrews and Hatfield, and Byford. At Woman's Hosp. Coll., 9 to 12—Profs. Marguerat, Bogue and Fitch; 3 to 6—Profs. Delafontaine, Curtis and Macdonald.

### FRIDAYS.

CLINICS. *Every Friday.*
At County Hospital, 2 P. M., *Medical*—Prof. Ross; 3 P. M., *Surgical*—Prof. Powell.
At Chicago College, 2 P. M., *Gynecological*—Prof. Roler.
At Mercy Hospital, 2 P. M., *On Dis. Eye and Ear*—Prof. Jones.

LECTURES. *Every Friday.*
At Rush College, 8½ to 12½—Profs. Gunn, Freer, Allen and Parkes; 4 to 6—Profs. Holmes and Etheridge. At Chicago College, 8½ to 12½—Profs. Hollister, Quine, Hyde and Bond; 3 to 6—Profs. Jones and Curtis, Andrews and Quine, and Roler. At Woman's Hosp. Coll., 9 to 12—Profs. Hotz, Bartlett and Blake; 3 to 5—Profs. Paoli and Stevenson.

### SATURDAYS.

CLINICS. *Every Saturday.*
At Rush College, 2 P. M, *Surgical*—Prof. Gunn; 3 P. M., *Diseases of the Brain and Nervous System*—Dr. Hay.
At Woman's Dispensary, 11 A. M., *Gynecological*—Drs. Hurlbut and Jackson; 1 P. M. *Electrical*—Dr. P. S. Hayes.
At Chicago College, 2 P. M., *Gynecological*—Prof. Nelson; *Surgical*—Prof. Andrews; 3 P. M.. *Medical*—Prof. Davis.

LECTURES. *Every Saturday.*
At Rush College, 8½ to 12½—Profs. Hay, Miller, Freer and Parkes. At Chicago College, 8½ to 12½—Profs. Hollister, Quine, Sherman and Hatfield; 3 to 6—Profs. Davis and Nelson, Andrews and Quine, and Byford. At Woman's Hosp. College, 9 to 12—Profs. Marguerat, Earle and Fitch; 4 to 6—Profs. Curtis and MacDonald.

☞ There are six special Clinics daily at the South Side Dispensary (Mercy Hospital) for students of the Chicago College. A daily Clinic at Dispensary of Hospital for Women and Children, 1½ P. M., for students of Woman's Hospital College.

